# Menstrual Disorders in Adolescence: Diagnostic and Therapeutic Challenges

**DOI:** 10.3390/jcm13247668

**Published:** 2024-12-16

**Authors:** Christiane Anthon, Marcel Steinmann, Angela Vidal, Carolin Dhakal

**Affiliations:** 1 OVA IVF Clinic Zurich, Clinic for Reproductive Medicine, 8005 Zurich, Switzerland; 2 Division of Gynecological Endocrinology and Reproductive Medicine, Women’s Hospital, Kantonsspital Lucerne, 6000 Lucerne, Switzerland; marcel.steinmann@luks.ch; 3 Division of Gynecological Endocrinology and Reproductive Medicine, University Women’s Hospital, Inselspital Bern, University of Bern, 3010 Bern, Switzerland; angela.vidal@insel.ch; 4 Fertisuisse, Clinic for Reproductive Medicine, 4600 Olten, Switzerland; carolina.nangpa-la@gmx.net

**Keywords:** menstrual disorders, adolescence, juvenile hypermenorrhea, high risk for polycystic ovary syndrome (PCOS), amenorrhea, hypogonadotropic hypogonadism, premature ovarian insufficiency, genital malformations, endometriosis

## Abstract

**Background**: Adolescence is the period of life between the ages of 10 and 19. This period is essentially dominated by puberty. The first menstruation, called menarche, occurs, on average, at the age of 12–13. The period after menarche, especially the first 2 years, is characterized by anovulatory cycles, which can be accompanied by menstrual irregularities. This review aims to describe the current status of the diagnostic and therapeutic challenges of the physiological and pathological causes of menstrual irregularities in adolescence and evaluates the benefits from interdisciplinary collaboration to ensure optimal care. **Methods**: A systematic literature search was conducted in the PubMed database in April 2024 using the following term: “menstrual disorder adolescence”. A total of 1724 abstracts were screened, and relevant articles from the last 10 years were included. In addition, a supplementary topic-relevant literature search of the guidelines of the European Society of Human Reproduction and Embryology (ESHRE) and the guidelines of the Arbeitsgemeinschaft der wissenschaftlichen medizinischen Fachgesellschaft (awmf) was carried out. **Results:** In addition to cycle irregularities that occur physiologically as a result of anovulatory cycles in the context of the immaturity of the hypothalamic–pituitary–gonadal axis, there are other cycle abnormalities that can be classified as pathological and need to be recognized and treated. **Conclusions**: Increasing awareness of the various specialist disciplines of physiological and pathological cycle abnormalities in adolescence and interdisciplinary cooperation between them can have a positive influence on the quality of life of adolescent women with cycle abnormalities.

## 1. Introduction

Adolescence is the period of life between the ages of 10 and 19 [1]. It is dominated by puberty, with its enormous physical and psychological changes and a particular vulnerability to emotional, moral, and intellectual development.

Menstrual health is essential for the well-being and quality of life of adolescents. In the period after menarche, girls are often insecure about this new function of their body and need help if any problems occur.

This review aims to describe the current status of the diagnostic and therapeutic challenges of the physiological and pathological causes of menstrual irregularities in adolescence and evaluates the benefits from interdisciplinary collaboration to ensure optimal care.

In addition to cycle irregularities that occur physiologically in the first 2 years after menarche due to the immaturity of the gonadal axis with anovulatory cycles [2,3,4], there are various cycle abnormalities that are considered pathological and should be recognized in time in order to provide girls with adequate treatment and monitoring.

## 2. Review

### Literature Search

**Figure 1:**
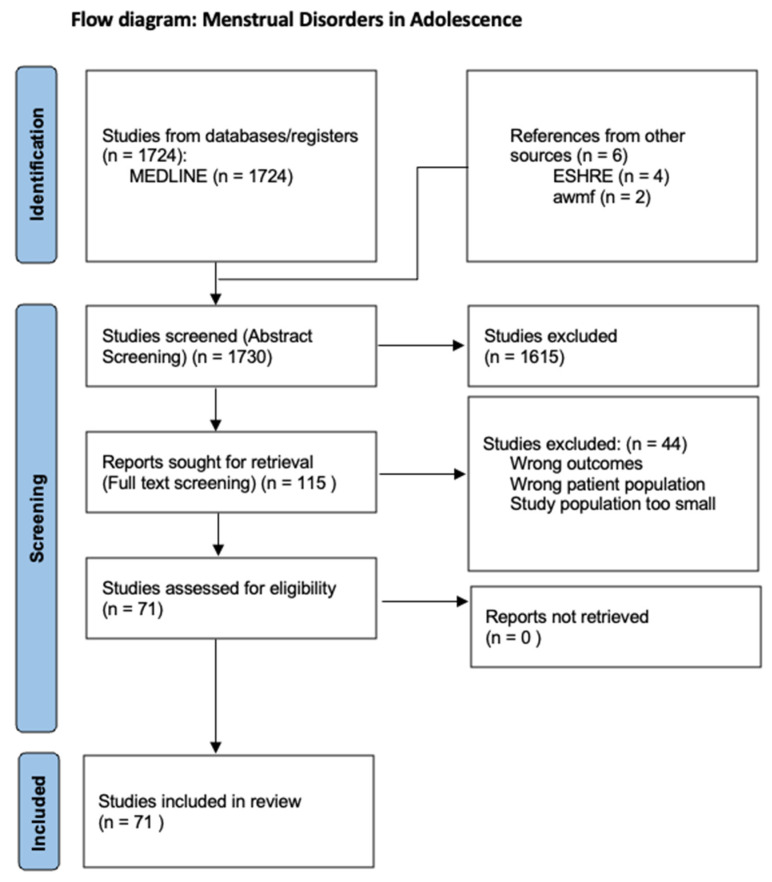
PRISMA flow diagram.

The aim of this review is to provide an overview of the various menstrual disorders in adolescence to differentiate these from menstrual disorders in adulthood and to describe their etiology and treatment. For this purpose, a systematic literature search was conducted in the PubMed database in April 2024 using the following term: “menstrual disorder adolescence”. All relevant articles from the last 10 years were included. Only English-language studies were included. The abstracts of 1724 articles were screened. Out of these 1724 abstracts, 115 articles were included in the full-text screening. These studies were analyzed narratively through content analysis. Of the 115 articles, 71 were included in the review (Figure 1). Cycle irregularities and disorders of various causes were the principal variables of interest. The inclusion criteria were studies of good quality related to patients in adolescence. Original articles that provided relevant information on menstrual irregularities or disorders in adolescence were included. In addition, a supplementary literature search of the relevant literature from the guidelines of the European Society of Human Reproduction and Embryology (ESHRE) and the guidelines of the Arbeitsgemeinschaft der wissenschaftlichen medizinischen Fachgesellschaft (awmf) was carried out.

## 3. The Onset of Puberty as a Transition from Childhood to Adolescence

Puberty begins with a change in the activity of the gonadotropin-releasing hormone (GnRH) neurons in the hypothalamus. The release of GnRH initially occurs at night, but as puberty progresses, it also pulsates during the day.

GnRH causes the release of luteinizing hormone (LH) and follicle-stimulating hormone (FSH) in the pituitary gland.

**Figure 2:**
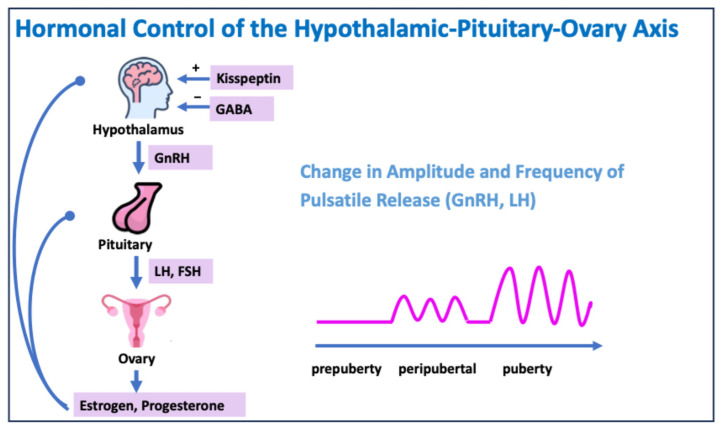
Hormonal control of the hypothalamic–pituitary–ovary axis. GABA: gamma–aminobutyric acid.

LH and FSH regulate the secretion of testosterone, estradiol, and progesterone in the gonads, which leads to the development of secondary sexual characteristics (Figure 2) [2].

## 4. Menarche and the Menstrual Cycle in Adolescence

Physical development during puberty lasts around 3 years and begins with thelarche, the growth of the mammary gland, at an average age of 11 years. On average, the first menstrual period, the menarche, occurs 2 years after the start of breast development. Menarche marks the end of the growth spurt.

Initially, the cycles are often anovulatory, and 2 years after menarche, up to 85% of the cycles are still anovulatory [3,5]. This means that follicle persistence and irregular cycles are considered physiological due to the immaturity of the gonadal axis. Ovulation occurs regularly at the end of puberty. Therefore, menstrual irregularities in the first 2 years after menarche do not need to be clarified, with the exception of disabling hypermenorrhea, anemia, or pain.

## 5. Special Features in Diagnostics

The usual investigations include a detailed medical history, a physical examination, a sonographic assessment of the internal genitalia, and an assessment of height and weight.

The examination of adolescent girls should be adapted to their stage of development and sexual activity. Each stage of pubertal development is categorized according to the Tanner stages. This makes it possible to assess whether the hormonal axis is functioning according to age and whether puberty is progressing properly.

**Figure 3:**
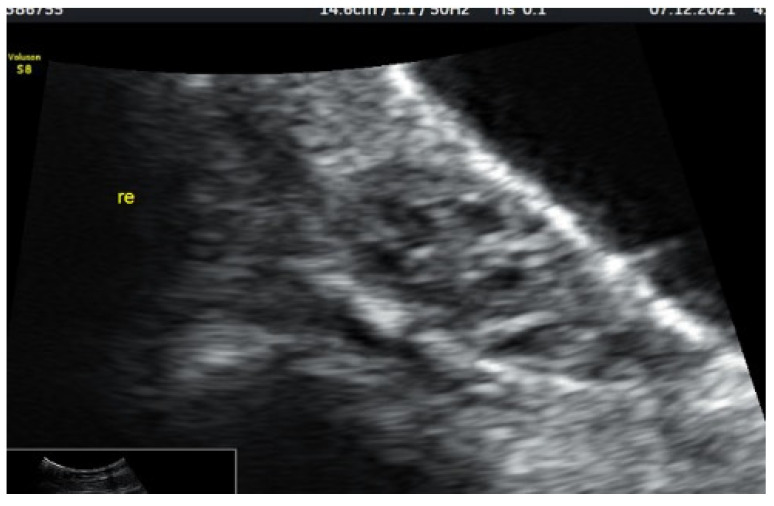
Abdominal sonography of an right (re) ovary in an adolescent girl (photo by the authors).

**Figure 4:**
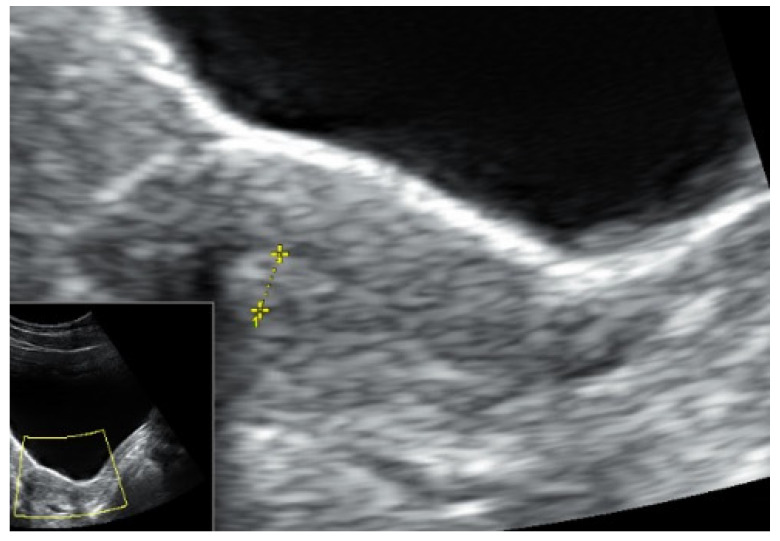
Abdominal sonography of the messurement of the tissue of the uterus in an adolescent girl (photo by the authors).

Inspection of the external genitalia is performed by the separation and traction of the labia majora, with discrete lateral or caudal traction. A pediatric speculum is rarely required for the examination. The sonographic assessment is performed by abdominal sonography with a full bladder (Figure 3 and Figure 4). The uterus and ovaries are assessed here, with the shape of the uterus providing information about the degree of estrogenization. Endometrial thickness, the antral follicles in the ovaries, and the presence of cysts or follicular persistence are also assessed. To answer specific questions, a genetic examination, magnetic tomography (MRI), and, rarely, laparoscopy can supplement the diagnosis. Certain situations make various functional tests useful: a GnRH (gonadotropin-releasing hormone) test to check pituitary function, an ACTH (adrenocorticotropic hormone) test in the case of congenital adrenal hyperplasia (CAH), a progestogen test to check whether there is sufficient endogenous estrogen activity and endometrial function in the case of amenorrhea, and an estrogen–progestogen test to assess the responsiveness of the endometrium in the case of uterine amenorrhea.

Hormone status determination, a bone age assessment, bone density measurements, an assessment of glucose metabolism, or specific examinations in cases of suspected chronic disease are reserved for special questions. Depending on the symptoms, pregnancy, sexually transmitted diseases, and trauma should be ruled out [6].

## 6. Menstrual Irregularities and Disorders in Adolescence

### 6.1. Dysfunctional, Juvenile Hypermenorrhea

Hypermenorrhea, defined as bleeding lasting longer than 7 days with a blood loss of more than 80 mL [7], is a common gynecological complaint among adolescent girls. Increased bleeding with large blood losses can have a negative impact on girls’ quality of life, preventing them from participating in social life, sports, or school, and is also associated with increased depression [8]. In addition, serious health problems can occur due to iron-deficiency anemia. Due to the immaturity of the hypothalamic–pituitary–ovarian axis, follicular persistence in anovulatory cycles occurs more frequently in the first few years after menarche as a cause of increased uterine bleeding [9]. This should be seen as part of normal pubertal development rather than a clinical issue [9]. The causes of continuous juvenile bleeding are the insufficient release of both GnRH and LH as well as a disruption in the feedback mechanism of the regulatory cycle, with an irregular rise and fall in estrogen levels and a prolonged estrogen phase or a disrupted estrogen–progestogen balance [5,9].

A differential diagnosis must consider an underlying hematological disease with an increased bleeding tendency, such as von Willebrand syndrome, a platelet function disorder, or a factor 7 or 8 deficiency [10,11,12]. If there is increased bleeding in the patient’s own or family history, this can be an additional indication of a coagulation disorder [9,11,13]. In 7–62% of cases, hypermenorrhea is caused by a coagulation disorder [14]. If a coagulation disorder is present, the treatment of hypermenorrhea is often not easy to manage. It requires interdisciplinary gynecological and hematological care in order to prevent recurrent hemoglobin (Hb)-effective bleeding [14,15]. The hemoglobin level should be determined in order to identify whether anemia is already present, as girls often find it difficult to subjectively assess the bleeding [16,17]. Symptoms of anemia, such as tiredness, fatigue, or dizziness, have an impact on quality of life [18]. Girls often do not present to a doctor or are not adequately diagnosed and treated [11].

**Figure 5:**
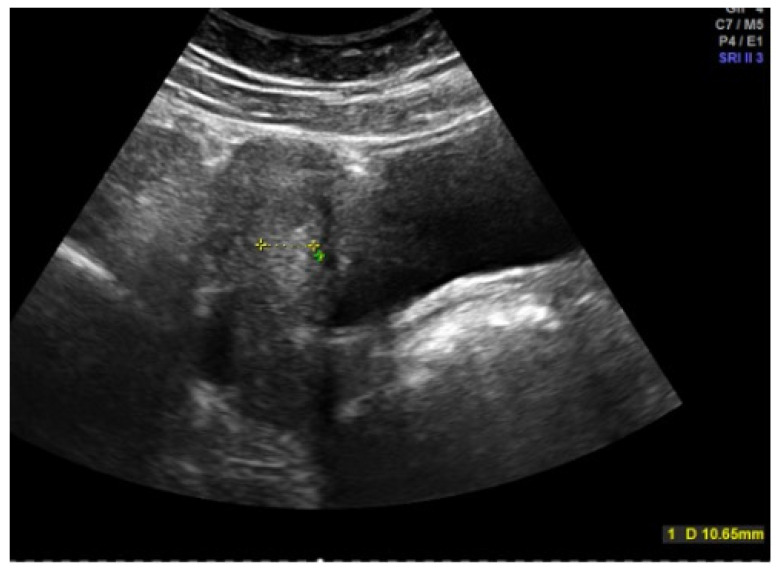
Abdominal sonography: Messurement of Excessively thickened endometrium in a 12-year-old girl with juvenile hypermenorrhea (photo by the authors).

Hormonal therapy is the treatment of choice [11,19]. After a sonographic assessment of the endometrial thickness (Figure 4 and Figure 5), progestogen monotherapy can be administered for 14 days (e.g., dydrogesterone 2 × 10 mg daily, medroxyprogesterone acetate 10 mg daily) in the case of an excessively thickened endometrium. If the endometrium is thin, there are various options. In this case, estrogenization of the endometrium with subsequent transformation must take place first. One option would be estrogen monotherapy for 10 days, followed by an estrogen–progestogen combination, as found in oral hormone replacement therapies. Another option, and the most commonly used, especially because of its prophylactic function, is an oral contraceptive [11]. In an acute situation, a high-dose contraceptive pill containing at least 30 µg of ethinylestradiol can be administered every 6–8 h until the bleeding intensity decreases, followed by a reduction to two and then one tablet daily if the bleeding cannot be stopped with the usual dosage [6]. The patient’s individual risk factors and contraindications must be taken into account. A progesterone-only pill can also be administered depending on the risk profile. A higher dosage of progestogens may also be necessary in the case of acute heavy bleeding, e.g., double the dose of desogestrel [5,6,20]. The third option is 2.5–5 mg of norethisterone acetate daily for 10 days, which, as a progestogen with an estrogenic partial effect, addresses both the thickening and transformation of the endometrium, but this is no longer freely available in all countries [16,21,22]. In rare cases, hospitalization may be necessary, especially if the hemoglobin level is already below 8 g/dL [6].

Both an oral contraceptive and hormone replacement therapy can be used for prophylaxis and, in the case of manifest anemia, until the hemoglobin level has recovered. In the case of hormone replacement therapy, the lack of contraceptive protection must be emphasized. Another effective option for patients with and without coagulation disorders is an IUD (intrauterine device) containing levonorgestrel [23,24,25]. Even with coagulation disorders, there is no increased expulsion rate [26]. The insertion of an IUD is often more difficult in adolescent women, especially if they have not yet engaged in sexual intercourse, and sedation may be necessary. In the case of heavy bleeding, iron substitution, blood transfusion, or tranexamic acid may also be necessary. Tranexamic acid is also important if girls refuse hormones or if there are contraindications [27,28]. Treatment of hypermenorrhea with tranexamic acid appears to be just as effective as treatment with hormones [28]. Surgical curettage should be avoided to protect the endometrium [8,16].

The aim of the therapy is to stop the bleeding, treat the anemia, and establish a regular cycle, thereby improving the quality of life [5].

### 6.2. Amenorrhea

There are many different causes of amenorrhea. A distinction is made between hormonal and organic causes and between primary and secondary amenorrhea. Primary amenorrhea is the absence of menarche until the age of 16. Secondary amenorrhea is the absence of menstruation for more than 90 days by someone who has had periods in the past [29].

Pregnancy should be ruled out at the beginning of any further diagnostics. If an already regular cycle becomes irregular again, the cause should be investigated.

Hypogonadotropic hypogonadism (WHO I) occurs with pubertas tarda. This condition is largely constitutional and has no pathological value, but disorders of the gonads, the hypothalamus, and the pituitary gland (for example, Sheehan syndrome), or the consequences of genetic defects (for example, Kallmann syndrome), can also be the cause [29].

Hypothalamic-pituitary dysfunction (WHO II) has two common causes in adolescence: a high risk of polycystic ovary syndrome and central regulatory dysfunction caused by stress, anorexia, or competitive sports.

Hypergonadotropic hypogonadism (WHO III), so-called premature ovarian insufficiency, is a condition defined by the loss of ovarian activity before the age of 40 years. It rarely occurs in adolescent women. However, approximately 1:10,000 patients under the age of 20 are affected. Possible causes include genetic factors such as Turner syndrome or a Fragile X premutation, an autoimmune predisposition, or iatrogenic causes following chemotherapy, radiotherapy, or ovarian surgery [30].

Purely organic causes of amenorrhea (WHO IV) are malformations of the genitals such as Meier–Rokitansky–Küster–Hauser syndrome or hymenal atresia [31]. The diagnosis is often only made due to primary amenorrhea or dysmenorrhea. The incidence of the various etiologies is described extremely heterogeneously and appears to vary a lot depending on the study and the underlying population. This is why no statement can be made [32,33].

A lack of follicular maturation leads to insufficient estrogenization and the thickening of the endometrium, so menstruation cannot take place. The effects of a lack of menstrual cycles with a consequent lack of estrogenization result in the absence of the onset of puberty and the development of secondary sexual characteristics as a short-term consequence. The long-term consequences of a lack of estrogenization include poor bone health and impaired cognitive and cardiovascular health.

Girls with both hypo- and hypergonadotropic hypogonadism, therefore, require a therapy that induces puberty and ensures normal estrogenization in the long term [31].

### 6.3. Hypogonadotropic Hypogonadism

Hypogonadotropic hypogonadism is the most common cause of both primary and secondary amenorrhea [34]. This is a functional disorder of the hypothalamus and gonadotropin release. In adolescent women, the causes are usually competitive sport, stress, or an eating disorder [34]. The level of sporting activity and body fat mass correlate with the likelihood of amenorrhea and the negative effects on bone density [35,36,37]. The so-called “female athlete triad” or, as the International Olympic Committee proposed, “relative energy deficiency in sport” describes a disorder characterized by low energy supply, cycle disorders, and low bone density [38]. Even calorie restriction within the normal BMI (body mass index) range can lead to a menstrual disorder, which is why eating disorders should be treated at an early stage to prevent long-term damage [39]. Rarer causes include brain tumors, craniocerebral trauma, perinatal asphyxia, radiotherapy of the brain, systemic diseases (e.g., histiocytosis, Wilson’s disease, thalassemia), or a genetic defect (e.g., Kallmann syndrome, septo-optic dysplasia, GnRH receptor defect) [40]. During the diagnosis, low-to-normal gonadotropin levels are found, with mostly low estradiol. Sonographically, a thin endometrium and ovaries that show no follicular maturation can be found. Even short-term food restriction leads to a prepubertal secretion pattern of gonadotropins, especially LH (Figure 2). The frequency of LH secretion is then too low for follicle maturation. This results in estrogen deficiency and cycle disorders, including amenorrhea [41]. Adequate estrogenization is important for the development of peak bone mass and long-term skeletal health [41]. Menstruation usually returns after approximately 6–12 months once normal weight has been reached. In around 10–30% of cases, amenorrhea persists despite weight gain. Bone density often takes years to recover or never reaches the appropriate peak bone mass, with a lifelong increased risk of fractures as a complication of osteoporosis [34,42,43]. Treatment should be multidisciplinary in collaboration with psychologists and psychiatrists. Bone density measurements should be carried out during the course of treatment to determine the current situation [44]. The best therapy is weight gain with the establishment of a normal menstrual cycle and adequate estrogenization. To achieve this, it may be necessary to reduce physical activity and increase calorie intake and BMI into the normal range [38]. Neurological and psychological development and health are also influenced by estrogenization [41]. In gynecological therapy, the focus is on estrogenization in order to minimize the long-term effects, particularly on bone health. Estradiol appears to have the best effect in this respect. However, it is particularly important that estrogenization is carried out, and alternatives such as contraceptive pills or a contraceptive patch (especially for patients suffering from bulimia) also have a positive effect. For endometrial protection, the endometrium must also be appropriately transformed with progesterone [44]. In addition, vitamin D and calcium should be supplemented. With a normal diet, calcium supplementation is not necessary, but with an eating disorder like anorexia nervosa, 1000–1500 mg should be supplemented daily [38,41,44].

### 6.4. Delayed Puberty/Pubertas Tarda

Delayed puberty, the absence of pubertal development and formerly known as “pubertas tarda”, is defined as the absence of breast development after around 13.5 years or a pause in pubertal development for more than 18 months. This occurs in 0.3% of adolescents. It is often a normal variant without pathological significance. The so-called constitutional delay in growth and development usually occurs in familial clusters [34]. However, if breast development has not started by the age of 13 and menarche has not occurred by the age of 16, the possible causes should be evaluated and puberty induction should be considered [45]. A family history of pubertal development can provide an indication of a constitutional developmental delay with familial clustering, with the possibility that puberty may just be delayed without pathological causes [45]. The older the girls are at the initial examination, the more likely it is that the cause is a pathological disorder. A differential diagnosis of delayed puberty due to competitive sports, malnutrition, drugs (such as cannabis), and chronic diseases (anorexia, chronic inflammatory bowel disease, cystic fibrosis, etc.) and malformations should be clarified, and disorders of the gonads, hypothalamus, and pituitary gland or genetic defects should be ruled out [34].

A clinical examination, determination of bone age, and exclusion of chronic diseases are recommended for diagnosis. In addition, a blood test to evaluate the thyroid gland, LH, FSH, estradiol, and prolactin should be performed. In constitutional developmental delay as well as in hypogonadotropic hypogonadism, LH and FSH with low values correspond to the child’s prepubertal hormone profile. In hypergonadotropic hypogonadism, LH and FSH are significantly elevated. Depending on the suspected diagnosis, an olfactory test, a GnRH test, an MRI of the brain, an abdominal ultrasound, and genetic tests may be helpful for an extended diagnosis.

Other causes of pubertas tarda can occur as part of syndromes such as Prader Willi or Leopard syndrome.

Nevertheless, the various causes of delayed puberty usually require the induction of puberty in order to ensure a normal phenotype and an adequate supply of estrogen. This is also recommended if, for example, there is still a likelihood of delayed development due to a chronic or mental illness in order to minimize the long-term consequences or improve well-being [45]. In general, puberty induction begins at the age of 11–13 years [30,45]. The estradiol dose is increased at 6-month intervals under close monitoring. After approximately 2 years, bleeding can then be initiated with progestogens, e.g., 200 mg of Utrogestan cyclically for 14 days [30,46] (Table 1). Table 1 provides an overview of the practical procedure for pubertal induction.

### 6.5. Premature Ovarian Insufficiency

Premature ovarian failure means the premature failure of ovarian function before the age of 40, which occurs with a prevalence of approximately 1% [30]. There are various underlying causes of the disease. A genetic cause can be a chromosomal disorder such as Turner syndrome, a Fragile X (FRAXA) premutation, XY gonadal dysgenesis, or androgen resistance [47].

An autoimmune cause leading to the destruction of the follicular pool may be indicated by an association with Addison’s disease or Hashimoto’s thyroiditis. Iatrogenic causes result, for example, from an underlying malignant disease that has been treated with chemotherapy, radiotherapy, or ovarian surgery with the destruction of the ovarian tissue. Premature ovarian insufficiency occurs very rarely in adolescence, affecting around 1:10,000 under 20-year-olds [30]. Evidence is provided by an elevated FSH level above 25 IU/L together with a cycle disorder in the form of oligo- or amenorrhea for at least 4 months [30]. FSH testing does not have to be timed to a specific day of the menstrual cycle, and assessment should be repeated after 4–6 weeks if there is diagnostic uncertainty [30]. For a more precise diagnosis, it is recommended that the karyotype, FRAXA premutation, 21-hydroxylase, and anti-Thyroid antibodies be determined. However, in a large number of those affected, the cause cannot be identified, and it is described as idiopathic POI [30]. Adolescent women often escape a precise diagnosis [48]. Depending on when the premature ovarian insufficiency occurs, it may be necessary to perform puberty induction so that secondary sexual characteristics develop. From the age of 11–13 years, low-dose 17-β-oestradiol should be started in increasing doses over a period of 2–3 years, as described in the ESHRE guidelines on premature ovarian insufficiency (Table 1) [46]. Close monitoring should be carried out every 3–6 months with an assessment of the Tanner stages, growth, and sonography of the internal genitalia. Estrogen accelerates bone maturation and could stop longitudinal growth, which is why there are controversial discussions about starting estrogen therapy. Low-dose estrogen can be started at the age of 12–13 years without losing height [30]. If Turner syndrome, especially Mosaic Turner syndrome, is present, this may not be recognized until adolescence. In Turner syndrome, a growth hormone is administered in childhood at the age of 4–6 years in order to achieve a better final height [46]. Girls with Turner syndrome do not have a growth spurt, which is normally initiated by endogenous estrogen [46]. The aim is to achieve an acceptable final height, bone mass gain, and normal psychosexual development to avoid psychosocial stigmatization. In girls with Turner syndrome, the long-term consequences of primary amenorrhea on bone mass were assessed, and a lower bone density with consequent increased fracture risk was found as a result of low estrogenization [49].

If the diagnosis is delayed, a modified regimen with a faster increase in estradiol can be used [30]. Transdermal administration is preferable because it results in more physiological estrogen levels with a lower risk of thrombosis [30,45].

Breast development usually begins after 6 months and is complete after 2 years. As soon as breakthrough bleeding occurs or after 2 years, regular transformation with progesterone should be started for endometrial protection and to prevent endometrial hyperplasia [30,46] (Table 1). After the completion of pubertal development, a maintenance dose of 100–200 µg of an estradiol-containing transdermal patch, 2 mg of estradiol gel, 2–4 mg of estradiol orally, or a combined contraceptive pill or hormone replacement therapy at an appropriate dose is recommended together with progesterone. The aim is to achieve an estradiol level equivalent to that of a regularly menstruating woman with an average of 180–370 pmol/L. Hormone replacement therapy should be used up to the average menopausal age of 51 years. Untreated premature ovarian failure is associated with a shortened life expectancy, mainly due to cardiovascular disease, which is why a balanced diet, exercise, smoking cessation, and the monitoring of cardiovascular risks are recommended [30].

Depression occurs more frequently, with estrogen deficiency causing greater vulnerability to it. If symptoms or sexual dysfunction occur in the context of vulvovaginal atrophy, treatment with local estrogenization can be carried out.

In addition to hormone replacement therapy, the most important pillar of therapy for bone health should be exercise and an adequate intake of calcium and vitamin D through diet or, if not possible, supplementation. Unfortunately, no interventions can increase ovarian activity [30].

Patients should be informed about the significance of the disease with regard to a later desire to have children. The spontaneous pregnancy rate for premature ovarian failure is around 5%. Only egg donation offers a real chance of pregnancy [30]. Nevertheless, the minimal probability of pregnancy should not be forgotten in order to avoid an unwanted teenage pregnancy due to the lack of contraception.

The anti-Müllerian hormone (AMH) value will be briefly discussed here in order to facilitate the assessment. The normal range of anti-Müllerian hormone (AMH) levels in childhood and adolescence is 0.7–7.5 ng/mL [50]. The peak is at the age of 25. Therefore, when AMH is low in adolescence, it must be recognized that this is a physiological range that has not yet peaked so as not to cause unnecessary concern for both girls and parents.

### 6.6. Hyperandrogenemia

Another group of cycle disorders results from hyperandrogenemia and the underlying causes. In adolescence, the following symptoms are caused by hyperandrogenemia: acne, hirsutism, alopecia, oligo-/amenorrhea, and specific symptoms such as pubertas praecox or clitoral hypertrophy. Rapid virilization indicates rare androgen-producing tumors. In obesity, androgens are mainly produced from fatty tissue, in addition to the physiological sites of origin, such as the adrenal cortex, the ovary, and the periphery.

Diagnostically, the first step here is to evaluate hormone status with an assessment of the androgens testosterone, androstenedione, DHEAS, and 17-Hydroxy-(OH)-progesterone (17-OHP) as well as the exclusion of a prolactinoma or hypothyroidism, which can be associated with hyperandrogenemia [51] (Figure 7). It is important to know that only the free androgens are effective.

### 6.7. Congenital Adrenal Hyperplasia (CAH)/Late-Onset Adrenogential Syndrome (AGS)

This is an autosomal recessive inherited disease due to a mutation of the 21-hydroxylase gene localized on chromosome 6 (approximately 90% of cases of congenital adrenal hyperplasia), which reduces enzyme activity by 30–50%. Most affected individuals have a compound heterozygous or homozygous mutation. One affected gene alone, a heterozygous mutation, does not cause the disease [52]. Approximately 4% of women with hyperandrogenemia have a 21-hydroxylase deficiency [52]. The 17-OH progesterone level is of particular importance in the diagnosis. If this is elevated above 2 ng/mL in the early cycle, CAH should be ruled out as the most important differential diagnosis of PCOS. The level of 17-OH-progesterone as a substrate of 21-hydroxylase increases if it is not working sufficiently or is absent. The inadequate function of 21-hydroxylase reduces cortisol production and leads to an increase in ACTH. This stimulates steroid hormone synthesis in the adrenal cortex in an unselected manner, i.e., also for the production of androgens [53].

If an elevated 17-OH progesterone >2 ng/mL is present, further diagnostics should be performed [52]. Firstly, a dexamethasone inhibition test to rule out Cushing’s syndrome as a differential diagnosis with similar symptoms is useful. Here, the suppression of cortisol is tested in the morning after administration of 1 mg of dexamethasone at 11 pm on the previous day. Another diagnostic step is an ACTH (adrenocorticotrophic hormone) test. Here, ACTH is administered intravenously. If there is an increase in 17-OH progesterone of more than 2.5 ng/mL, a genetic test should be carried out to assess whether a 21-hydroxylase deficiency is present (Figure 7). Other mutations, such as 11-β-hydroxylase deficiency, a 3-β-hydroxysteroid dehydrogenase defect, and others, can also lead to CAH but are far less common. Girls are asymptomatic for a long time. Symptoms can range from premature pubarche, tall stature, and bone acceleration to clitoral hypertrophy and the usual symptoms of androgen excess, such as hirsutism, acne, alopecia, oligo-amenorrhea, and infertility [52,53]. The significance of congenital adrenal hyperplasia (CAH) is particularly important with regard to the later desire to have children. If the partner is also affected by a mutation, there is a risk that the child will be homozygous for a severe mutation with low or hardly any enzyme activity and will be born with severe CAH with salt wasting. In girls, this can be accompanied by genital malformations in the sense of virilization. This should be avoided at all costs by having both partners diagnosed, discussing the issue with them, and, if necessary, administering cortisol therapy during pregnancy, as it may be difficult or impossible to completely correct the condition later on, depending on its severity. Otherwise, glucocorticoid therapy would only be recommended in the case of puberty praecox or the acceleration of bone age [52].

Cycle regulation with an anti-androgen pill is used therapeutically, which treats both the symptoms of hyperandrogenemia and the cycle disorder by reducing androgens. Follicular stimulation is helpful if the patient wishes to have children, as there is often insufficient ovulation [53].

### 6.8. High Risk for Polycystic Ovary Syndrome (PCOS)

Another cause of cycle disorders in adolescence is caused by a so-called high-risk constellation of symptoms for polycystic ovary syndrome. According to the Rotterdam criteria for the diagnosis of PCOS syndrome, the diagnosis is considered confirmed in adulthood if two of the following three diagnostic criteria are met:

- Oligo-/amenorrhea;
- Hyperandrogenemia or androgenization symptoms;
- Sonomorphological signs of polycystic ovaries.

These criteria cannot be used in adolescence. PCOS should not be diagnosed until 8 years after menarche [51]. What further complicates the diagnosis in adolescence is that the androgen concentrations in the blood only reach the level of adult women at the age of 12–15 [53], and there are no clearly defined cut-off values [54]. Androgens are produced in the adrenal cortex, periphery, and in the adipose tissue.

Sonomorphological signs of polycystic ovaries are not suitable as a diagnostic criterion for adolescent women [54], as around 33% of girls in this age group have sonographically polycystic ovaries. Therefore, a polycystic ovary in adolescence does not yet constitute PCOS [55]. Oligomenorrhoea and hyperandrogenemia appear to indicate an increased risk of developing PCOS later in life [55,56,57]. Adolescent women are therefore classified as high risk for PCOS and monitored closely without immediately stigmatizing and unsettling them with a PCOS diagnosis. Nevertheless, due to the high risk, individualized monitoring should be carried out, as comorbidity with an increased risk of metabolic syndrome with impaired glucose tolerance, type II diabetes mellitus, dyslipidemia, arterial hypertension, sterility, and an increased risk of endometrial cancer or depression are known [51].

**Figure 6:**
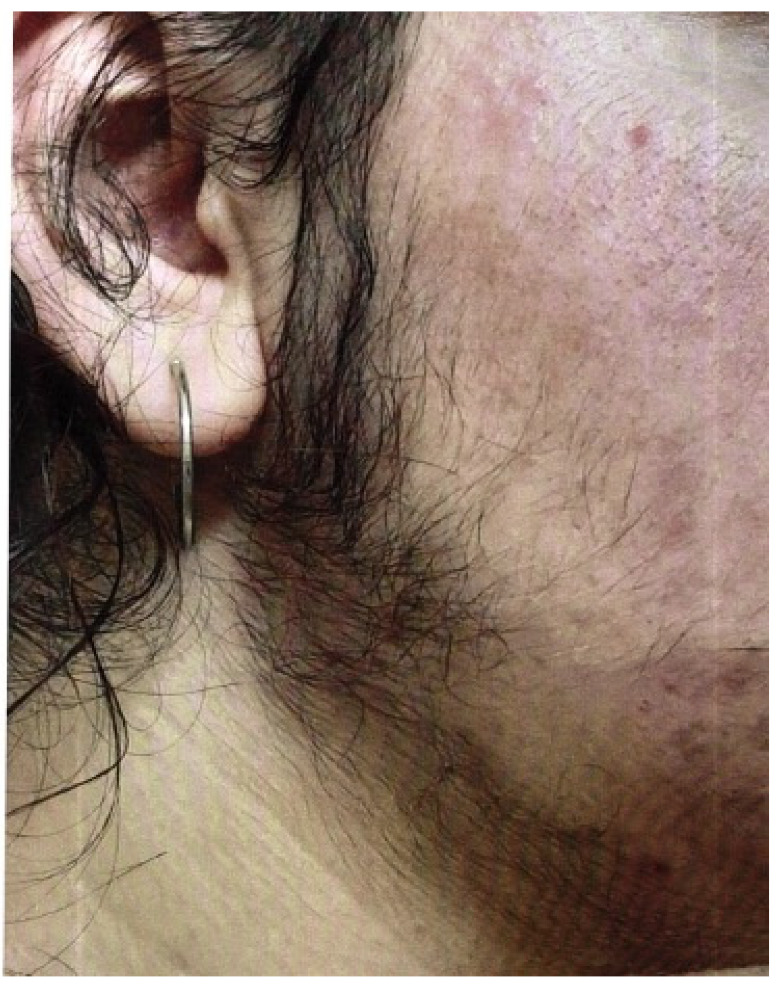
A 16-year-old girl with pronounced hirsutism and at high risk for PCOS (photo by the authors).

The Ferriman–Gallwey score can be used to assess hirsutism, in which patients assess their own body hair. Figure 6 shows a 16-year-old girl with pronounced hirsutism and obesity permagna due to a high-risk constellation of symptoms for PCOS.

**Figure 7:**
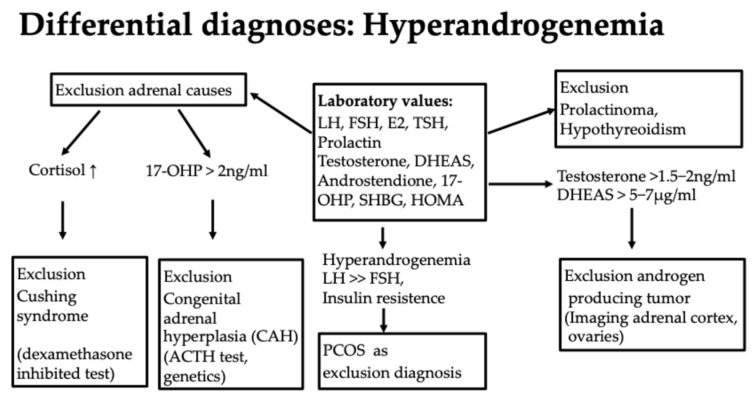
Differential diagnoses of hyperandrogenemia.

In addition to hyperandrogenemia, patients can often show other typical laboratory abnormalities, such as an LH/FSH quotient >>2, a pathological glucose metabolism with a Homa index >2.5, a pathological oral glucose tolerance test, or an abnormal HbA1c. The level of 17-OH progesterone is normal, which differentiates it from congenital adrenal hyperplasia (CAH); the AMH (anti-Müllerian hormone) level is elevated. In obesity, the SHBG value is often low, or dyslipidemia is present. However, these laboratory abnormalities are not among the diagnostic criteria [58]. The diagnosis is always a diagnosis of exclusion, in contrast to the other differential diagnoses of hyperandrogenemia (Figure 7). The etiology remains largely unknown, although both genetic and environmental factors appear to play a role. Pathophysiologically, several mechanisms lead to anovulation in PCOS—a “two-hit” theory is often discussed, in which a predisposition must come together with a provoking factor, such as insulin resistance [55]. The treatment of PCOS should be approached in a multidisciplinary manner. Cycle disorders are primarily treated with an anti-androgen contraceptive pill [54]. Oral contraception is also the treatment of choice for acne, hirsutism, and regular endometrial transformation in amenorrhea [54]. Cyproterone acetate, dienogest, chlormadinone acetate, and drospirenone are considered potent antiandrogenic progestins. The reduction in high androgen levels thus leads to an improvement in acne and hirsutism. However, patience is required when it comes to the effect on hair growth; it can take a good 6–9 months for success to become apparent. In the case of obesity, a lifestyle change should be discussed, with exercise and nutritional counseling being important pillars [51]. Cooperation with dieticians or an obesity center is often helpful, and multidisciplinary care improves the success of the treatment. Metformin, as an insulin sensitizer, can be used to support weight reduction. It is particularly effective in cases of insulin resistance and BMI over 25 kg/m^2^. It is dosed gradually to a maximum of 2000 mg daily to minimize gastroenteric side effects such as diarrhea. However, this therapy only works in combination with exercise [51]. Support from a dermatologist can be helpful in the treatment of acne. In the case of hirsutism, which can be perceived as very stressful, laser therapy can be tried. Hair growth on the face can be slowed down with eflornithine cream so that it is not necessary to shave as often. Oral contraception may be contraindicated in the case of pronounced obesity or additional risk factors such as a coagulation disorder related to thrombophilia, arterial hypertension, or nicotine abuse. In this case, a trial with progestogen-only preparations, such as cyproterone acetate or drospirenone, can be undertaken. Individual needs, such as the need for contraception, should not be forgotten. Amenorrhea poses a certain risk to the endometrium, as no transformation takes place, and endometrial hyperplasia could develop in this context. The increased risk of metabolic syndrome makes close monitoring with OGTT sensible, as well as the monitoring of weight and blood pressure, even in slim girls, and lipid checks, in order to refer girls for treatment at an early stage if there is a corresponding risk [51].

### 6.9. Dysmenorrhea with Endometriosis and Genital Malformations

Genital malformations and endometriosis, usually resulting in dysmenorrhea, will only be discussed in a brief digression for the sake of completeness.

### 6.10. Endometriosis

Dysmenorrhea is defined as painful menstrual bleeding. It is distinct from primary dysmenorrhea, which is painful menstruation in the absence of specific pathologic conditions, and secondary dysmenorrhea, which is painful menstruation in the presence of pathologic conditions caused by a reproductive system disorder that leads to menstrual cramps, such as endometriosis or fibroids. Endometriosis occurs in approximately 2% of adolescent women [59].

Endometriosis is defined as an estrogen-dependent, inflammatory disease with a functioning endometrial mucosa outside the uterine cavity. It is a likely diagnosis in cases of refractory pain, acyclic lower abdominal pain, dysmenorrhea, dysuria, dyschezia, and dyspareunia [59,60]. In adolescent women, increased absenteeism from school or sports can also be interpreted as an indication [61,62]. The pain is also often accompanied by gastrointestinal complaints such as nausea, vomiting, dyschezia, and constipation or vasovagal reactions. If the diagnosis is endometriosis, which can only be confirmed by histological confirmation, the first line of treatment is conservative medication. Analgesia with non-steroidal anti-inflammatory drugs (NSAIDs) and hormonal therapy with progestogens or an extended-use contraceptive pill (off-label use) are available to minimize the progression of the disease until the patient wishes to have children [63]. GnRH analogs should be used very cautiously in adolescence due to their negative effects on bone, with a long-term risk of osteoporosis [64]. If symptoms persist, surgical treatment must also be considered, but this should always be performed at a center with a high level of expertise in order to preserve the ovarian reserve [64,65]. In adolescence as well as in adulthood, there appears to be a delay in diagnosis, and patients are often not treated adequately [65,66]. One of the reasons may be that the detection rate on ultrasound is low [63], and surgery is very reluctantly indicated in adolescent women. However, treatment can also be justified without histological confirmation based on the clinical findings, and girls benefit from this.

### 6.11. Genital Malformations

The main symptom of genital malformations is primary amenorrhea [31]. Genital malformations that lead to pain are mainly hymenal atresia and a uterus didelphys with a vaginal septum that closes a horn, as well as a rudimentary uterine horn with an endometrium that bleeds periodically [67]. In summary, any form of malformation that obstructs the outflow of menstrual blood leads to dysmenorrhea. Directional signs can also be a problem when using a tampon.

Hymenal atresia usually occurs in isolation and is not associated with higher malformations. On examination, the vagina is closed, and there may be a hematocolpos or hematometra [31,68].

Treatment depends on the exact malformation. Malformations associated with recurrent pain symptoms should be surgically repaired [67,69].

Mayer–Rokitansky–Küster–Hauser syndrome, which occurs with an incidence of 1:5000, is often not recognized in childhood and is characterized by primary amenorrhea.

This is an inhibition malformation of the Müller ducts that leads to uterine aplasia and is associated with renal and skeletal malformations. Therapeutically, the reconstruction of a neovagina is useful here in order to enable patients to have largely normal sexuality [31,70].

To assess a genital malformation, the external genitalia should be inspected, an abdominal ultrasound with a full bladder performed, a renal ultrasound performed, and hormones determined to exclude hormonal causes. An MRI would be the first-line diagnostic procedure for suspected malformation in adolescence [71]. Diagnostic laparoscopy can be performed as a further diagnostic procedure if necessary [31].

Once the diagnosis has been made, a decision about whether surgical treatment is necessary can be made, and this can be initiated.

## 7. Conclusions

To summarize, it can be said that there are various causes of cycle disorders in adolescence. In contrast to cycle disorders in adulthood, cycle irregularities often occur in the 2 years after menarche due to anovulatory cycles and the immaturity of the hypothalamic-pituitary–ovarian axis without having a pathological value. The resulting anemia or dysmenorrhea always requires treatment or clarification. Because of the various causes of cycle disorders, it is important to find out what the underlying cause is so that adolescent women receive the appropriate treatment and check-ups. However, care should be taken to ensure that adolescent women are not stigmatized and unsettled by a premature diagnosis. When treating adolescent girls, we have to keep in mind that there is a gap in treatment when they are too old to visit a pediatrician but may not feel old enough to see a gynecologist. In this gap, it is very important that the family doctor keeps in touch with them, is aware of the issue, and works hand in hand with clinicians of other disciplines. The most important role for a pediatrician, gynecologist, family doctor, or hematologist is to support adolescent girls with cycle irregularities to improve their quality of life and prevent serious health problems through interdisciplinary collaboration.

This review summarizes the current knowledge on the diagnosis and treatment of menstrual disorders in adolescents. There are still gaps in the current knowledge, for example, about blood test reference ranges, drug dosages, and alternative treatment options. More research is needed to close these gaps.

## Figures and Tables

**Table 1:** Puberty induction/estrogen replacement in adolescence, modified according to the ESHRE guidelines on premature ovarian insufficiency (POI) [30].

Age	Age-Specific Suggestions	Dose
12–13 years	No spontaneus development, and FSH is elevated	17-β-estradiol (E2) Transdermal: 6.25 µg/day via patch Oral: 5 µg/kg/day or 0.25 mg/day
12.5–15 years	Increase E2 dose at 6–12 month intervals over 2–3 years to adults’ dose	Transdermal: 12.5, 25, 37.5, 50, 75, 100 µg/day (adult dose: 100–200 µg/day) Oral: 5, 7.5, 10, 15 µg/kg/day (adult dose: 2–4 mg/day)
14–16 years	Cyclic progesterone after 2 years or when breakthrough bleeding occurs	Oral micronized progesterone 100–200 mg/day or dydrogesterone 5–10 mg/day during 12–14 days of the month
